# Low Night Temperature Affects the Phloem Ultrastructure of Lateral Branches and Raffinose Family Oligosaccharide (RFO) Accumulation in RFO-Transporting Plant Melon (*Cucumismelo* L.) during Fruit Expansion

**DOI:** 10.1371/journal.pone.0160909

**Published:** 2016-08-08

**Authors:** Jinghong Hao, Fengying Gu, Jie Zhu, Shaowei Lu, Yifei Liu, Yunfei Li, Weizhi Chen, Liping Wang, Shuangxi Fan, Cory J. Xian

**Affiliations:** 1 Beijing Key Laboratory of New Technique in Agricultural Application, College of Plant Science and Technology, Beijing University of Agriculture, Beijing 102206, China; 2 Institute of Agro-products Processing Science and Technology, Chinese Academy of Agricultural Sciences, Beijing 100193, China; 3 Institute of Protected Horticulture, Chinese Academy of Agricultural Engineering, Beijing 100125, China; 4 Key Laboratory of Protected Horticulture of Education Ministry and Liaoning Province, College of Horticulture, Shenyang Agricultural University, Shenyang 110866, China; 5 Beijing Agricultural Technology Extension Centre, Beijing 100029, China; 6 Sansom Institute for Health Research, School of Pharmacy and Medical Sciences, University of South Australia, Adelaide 5001, Australia; Universidade Federal de Vicosa, BRAZIL

## Abstract

Due to the importance and complexity of photo assimilate transport in raffinose family oligosaccharide (RFO)-transporting plants such as melon, it is important to study the features of the transport structure (phloem) particularly of the lateral branches connecting the source leaves and the sink fruits, and its responses to environmental challenges. Currently, it is unclear to what extents the cold environmental temperature stress would alter the phloem ultrastructure and RFO accumulation in RFO-transporting plants. In this study, we firstly utilized electron microscopy to investigate the changes in the phloem ultrastructure of lateral branches and RFO accumulation in melons after being subjected to low night temperatures (12°C and 9°C). The results demonstrated that exposure to 9°C and 12°C altered the ultrastructure of the phloem, with the effect of 9°C being more obvious. The most obvious change was the appearance of plasma membrane invaginations in 99% companion cells and intermediary cells. In addition, phloem parenchyma cells contained chloroplasts with increased amounts of starch grains, sparse cytoplasm and reduced numbers of mitochondria. In the intermediary cells, the volume of cytoplasm was reduced by 50%, and the central vacuole was present. Moreover, the treatment at 9°C during the night led to RFO accumulation in the vascular bundles of the lateral branches and fruit carpopodiums. These ultrastructural changes of the transport structure (phloem) following the treatment at 9°C represented adaptive responses of melons to low temperature stresses. Future studies are required to examine whether these responses may affect phloem transport.

## Introduction

Phloem is responsible for the transport of photoassimilates, such as carbohydrates, amino acids, and other nutrients from source tissues such as mature leaves to sink tissues such as fruits, shoots and root apical tissues, and developing organs [1–3]. Hormones, mRNAs, small RNAs and proteins are also transported by the phloem. Thus, the phloem performs a number of distinct tasks including collecting metabolites and signals from source organs, transporting these molecules and then releasing them to sink organs. Therefore, the phloem has significant potential function in facilitating inter-organ communication/coordination and hence in promoting plant growth and development [4].

The functions of the phloem are different according to the organ’s locations. While the loading phloem in source organs (minor veins) is responsible for the active loading of photo assimilates into the phloem, the unloading phloem in sink organs delivers assimilates to sink tissues, and the transport phloem (generally found in the main leaf veins of petioles, stems and primary roots, along the path from sources to sinks) translocates water and solutes from sources to sinks [5]. Active retrieval and the release of solutes (lateral transport) also occur through carriers of the sieve element (SE) plasma membrane along the transport phloem [6,7]. By this way, the transport phloem provides the surrounding tissues with assimilates for growth, and it is plausible that changes in the phloem ultrastructure of the lateral branches may bring about differences in the metabolic energy for the long-distance transport.

Unlike sucrose-transporting plants such as *Arabidopsis* and tobacco, raffinose family oligosaccharide (RFO)-transporting plants such as melons (belonging to the family Cucurbitaceae) translocate significant concentrations of RFOs in their phloem, primarily stachyose [8,9]. Their phloem cells are different from those of sucrose-transporting plants, and parts of their companion cells (CCs) develop specialized CCs known as intermediary cells (ICs) only in minor veins. Due to the complexity of RFO-transporting plants such as melon, cucumber, and Verbascum phoeniceum, it is important to study the characteristics of stachyose metabolism, phloem cells and long-distance transport, as well as their responses to environmental stimuli and challenges.

Low temperature is a major factor that limits the geographical locations suitable for plant growth and periodically accounts for significant losses in plant production. Melon (*Cucumis melo* L.) is a global crop in terms of economic importance and nutritional quality. It was originated from Africa and tropical regions in Asia and hence is sensitive to chilling temperatures. Its protected cultivation in winter, late autumn and early spring in temperate regions such as China is, however, usually subjected to low temperatures, especially low night temperature of about 10°C during fruit development, leading to an altered plant metabolism and a significant loss of productivity.

Currently, while investigations on alterations in photo assimilate production [10], carbohydrate metabolism [11,12], gene expression related to phloem-loading features, the location and phloem cell morphology [13,14] have been carried out in the seedlings of some RFO-transporting plants responding to low temperatures, studies on other stages of development of these plants have been limited. To gain more comprehensive information for low temperature effects on plants, we have recently selected melon plants which were at fruit developing stage and have found that low night temperatures significantly inhibited photosynthetic capacity [15], expression of galactinol synthase genes (CmGOS), and stachyose metabolism. Low night temperatures were also found to lead to mass accumulation of carbohydrates particularly raffinose (with the ratio of raffinose to total carbohydrates exhibiting a large increase) in melon leaves, indicating that phloem loading might be repressed [16]. Moreover, we found that low night temperatures decreased the carbohydrate content in fruits and affected the sink strength [15,17]. However, to obtain a better understanding of the carbon allocation between the sources and the sinks under cold stress, it is important to investigate potential changes in the phloem ultrastructure of lateral branches which connect source leaves and the sink fruits for the long-distance transport, and to examine whether these potential phloem cellular changes affect carbohydrate transport. However, reports related to these topics are few or almost non-existent.

Therefore, the objective of this work was to investigate the changes in the phloem ultrastructure of lateral branches in an RFO loading plant, i.e., melon, in response to low night temperatures (12°C and 9°C). This study represents the first time investigation, utilizing transmission electron microscopy, to observe changes of cell ultrastructure in the phloem of lateral branches and RFO accumulation in response to low night temperatures of 12°C and 9°C. Results from this study will provide a basis for future further studies probing into the interaction between source leaves, transport phloem, and sink fruits and the impact of cold stress.

## Materials and Methods

### Plant materials and culture conditions

Seeds of the cultivar Yumeiren (*Cucumis melo* L.), a popular pellicle melon variety from Northeast China, were sown in a sand/soil/peat (1:1:1 v/v) mixture and were then cultivated for 30 d under glass greenhouse conditions (12 h light, 300–1300 μmol/(m^2^ s), 26 ± 2°C during the day and 15 ± 2°C at night, 12 h dark, 50%-70% relative humidity). The seedlings were transferred into 25 cm × 25 cm plastic buckets containing a mixture of organic matter of soil (2:1 v/v) with NPK fertilizer and were maintained in the controlled environment described above. The plants were watered and fertilized by normal cultivation management. In brief, the plants were watered when the surface of nutrient medium was little dry and they were fertilized in the periods of anthesis and fruit expansion. The female flowers were hand-pollinated and tagged at anthesis, and the fruit load was limited to one per plant at the 12^th^ to 14^th^ node.

### Low night temperature stress treatments

Following pollination for 7 d and after the young fruit had started to expand, the plants were environmentally hardened in a controlled environmental chamber to receive different night temperature treatments [12 h light; 6:00–10:00 (475±50) μmol/(m^2^ s), 10:00–16:00 (1045±50) μmol/(m^2^ s), 16:00–18:00 (570±50) μmol/(m^2^ s); 26°C; 12 h dark at 15°C, 12°C and 9°C; 15°C was used as the control, with 60% relative humidity]. The experiment of low night temperature treatments was performed in three replicates.

**Fig 1:**
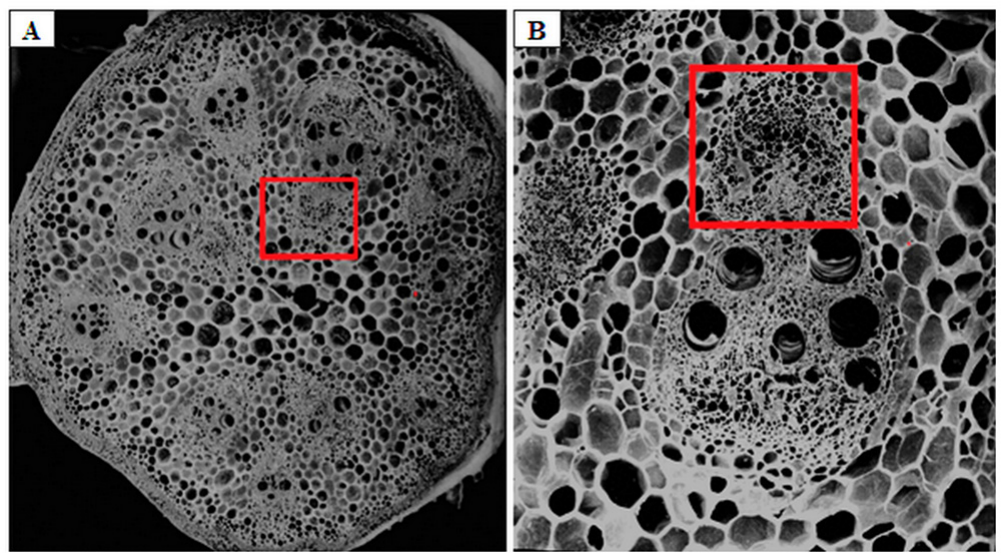
Transverse section of melon lateral branches. (A) Transverse section of melon lateral branches under normal night temperature conditions; (B) A vascular bundle in lateral branches.

At day 12 of the low night temperature treatments, small lateral branches on which fruits were growing, about 1–2 mm in length, were collected from control plants for processing for cross-sections for scanning electron microscopy (SEM) examination. The small phloem fragments of the lateral branches in the same position (marked by red box shown in Fig 1), approximately 1–2 ×1–2 mm, were collected from control and treatment plants for transmission electron microscopy (TEM) observations. Meanwhile, the lateral branches and carpopodiums were collected and frozen in liquid nitrogen for analyzing the content of RFOs (including stachyose and raffinose in this experiment).

### Scanning electron microscopy (SEM)

The samples were fixed in 2.5% glutaraldehyde in 0.1 M cacodylate buffer (pH 7.2) overnight at 4°C with three changes, dehydrated in an graded ethanol series, critical-point-dried through carbon dioxide, mounted on stubs, and coated with gold [18]. The preparations were observed with an S-450 scanning electron microscope.

### Transmission electron microscopy (TEM)

The sample fragments were fixed in 2.5% glutaraldehyde overnight at 4°C with three changes. The phloem of a single vascular bundle was excised from the same region of each lateral branch, collected, washed with 0.1 M phosphate buffer and fixed with 1% osmiumtetroxide overnight at 4s°C. The phloem tissues were then washed again with 0.1 mol·L^-1^ phosphate buffer, dehydrated in a graded series of ethanol and acetone solutions (30%, 50% and 70% ethanol for 15 min each; 80% and 90% acetone for 15 min each; and 100% acetone 3times for 15 min each), infiltrated with acetone and resin mixtures at proportions of 3:1, 1:1 and 1:3 and then embedded in resin. After polymerization (37°C for 24h, followed by 45°C for 24 h, and 60°C for 24 h), the embedded tissues were sectioned with a LKB-2088 ultramicrotome, stained with uranyl acetate and lead citrate followed by Reynolds lead citrate [19]. Stained sections were then observed and imaged using a JEX-100CX II transmission electron microscope.

### RFO content analyses

The samples of lateral branches and carpopodiums were ground in 10 mL 80% (v/v) ethanol in a tube, and then the tube was placed in a boiling water bath for 1 h, cooled, and centrifuged at 1,000 g for 10 min. The pellet was extracted two additional times with 10 mL 80% (v/v) ethanol. The supernatants from each extraction were combined and evaporated to dryness in a boiling water bath. The samples were resolubilized in 0.5 or 1 mL distilled water and filtered through an acetate filter (0.45 μm pore size, Nalgene, Thermo Fisher Scientific, Waltham, MA).

The contents of RFOs (including stachyose and raffinose in this experiment) were determined using the method of high performance liquid chromatography (HPLC) [19]. The system included a Waters 6000A pump (Millipore, Waters Chromatography Division, Milford, MA), an Inertsil NH_2_ column (250 mm×4.6 mm, 5 μm, Dikma Company, Forest Lake, CA) and a Waters 2410 refractive index detector connected to a strip chart recorder. Distilled water, at a flow rate of 10 mL/min, was used as the solvent of the 70% (v/v) acetonitrile. The column temperature was maintained at 35°C and was preceded by a Waters Bondapak C_18_/Corasil guard and a set of anion and cation cartridges (deashing guards, Bio-Rad Laboratories, Richmond, CA). All guards were operated at an ambient temperature of 25°C, and 20 μL samples were injected. The RFOs were identified and quantified from the retention times and the peak heights of stachyose and raffinose standards. All of chemicals were of chromatographical grade in purity. The standards of stachyose and raffinose were purchased from Sigma (St. Louis City, MO).

### Statistics and data analysis

The experiment was performed in three replicates, and each replicate has three samples (from three plants). For the analyses of TEM images, each replicate has three samples (from three plants), fifty visual fields were observed for each sample and about one hundred cells were analyzed. The percentages of certain phenotypes were the ratios of the numbers of phenotype cells to the total of one hundred cells in the fifty visual fields (including plasma membrane invagination in companion cells, plasma membrane invagination in intermediary cells, expanded central vacuole in intermediary cells, and the cytoplasm forming strongly stained and thinner annular rings in intermediary cells). The results shown in Table 1 represent the means of three replications (with each replication being a pool of 3 plants). The numbers of starch grains in intermediary cells or in phloem parenchyma cells or the numbers of mitochondria in phloem parenchyma cells were also obtained in the fifty visual fields (about one hundred cells) in each sample. The percentages in low night temperature treatments were calculated compared with control (considered as 100%). Data of RFO contents represent the means ±SD of three replications and each replication had 3 plants. Data were statistically analyzed using analysis of variance (ANOVA) by SPSS 10.0 (International Business Machine, Chicago, IL). The least significant difference (LSD) was calculated for the significantly different data at the 0.05 or 0.01 significance levels. Figures representing the physiological parameters were drawn using OriginPro (OriginLab Corporation, Northampton, MA).

**Table 1:** Changes in cell morphology of the phloem ultrastructure of lateral branches of melons after 12 day of low night temperature treatment at 12°C or 9°C when compared with control treatment at 15°C.

Changes	15°C	12°C	9°C
Plasma membrane invagination in companion cells	0 cell A	38 cells, 38%±0.02 B	99 cells, 99% ±0.01 C
Plasma membrane invagination in intermediary cells	0 cell Aa	0 cell Aa	99 cells, 99%±0.01 Bb
Expanded central vacuole in intermediary cells	8 cells, 8% ±0.02 Aa	96 cells, 96%± 0.01 Bb	99 cells, 99% ±0.01 Bb
The cytoplasm formed a strongly stained and thinner annular ring in intermediary cells	8 cells, 8% ±0.02 Aa	96 cells, 96% ±0.02 Bb	99 cells, 99% ±0.02 cells Bb
The number of starch grains in intermediary cells	50, 100% Aa	50, 100% Aa	i 60, 120%±0.32 Bb
The number of starch grains in phloem parenchyma cells	150, 100% A	620, 413%±0.52 B	1092, 728% ±0.81 C
The number of mitochondria in phloem parenchyma cells	750, 100% A	713, 95%±0.022 B	638, 85%±0.041 C

Note: Fifty visual fields and about one hundred cells were observed in each sample, and the precise numbers and percentages of cells with certain phenotypes are given. These numbers and percentages were means (± standard errors) from 3 replications (with each replication being a pool of 3 plants). The different small letters (a, b, c) represent significant difference at 0.05 level, and the different capital letters (A, B, C) represent significant difference at 0.01 level.

## Results

### SEM analyses of phloem in the lateral branches of melons

The vascular bundles of the melons consisted of inner and outer rings, with each ring containing five vascular bundles (Fig 1A). The vascular bundles showed a bicollateral structure, in which the phloem was located on both the inner and outer sides of the xylem, organizing it into internal and external phloem (Fig 1B). In the subsequent studies, the internal phloem was observed using TEM.

### TEM analyses of lateral branch phloem ultrastructure prior to low night temperature treatments

**Fig 2:**
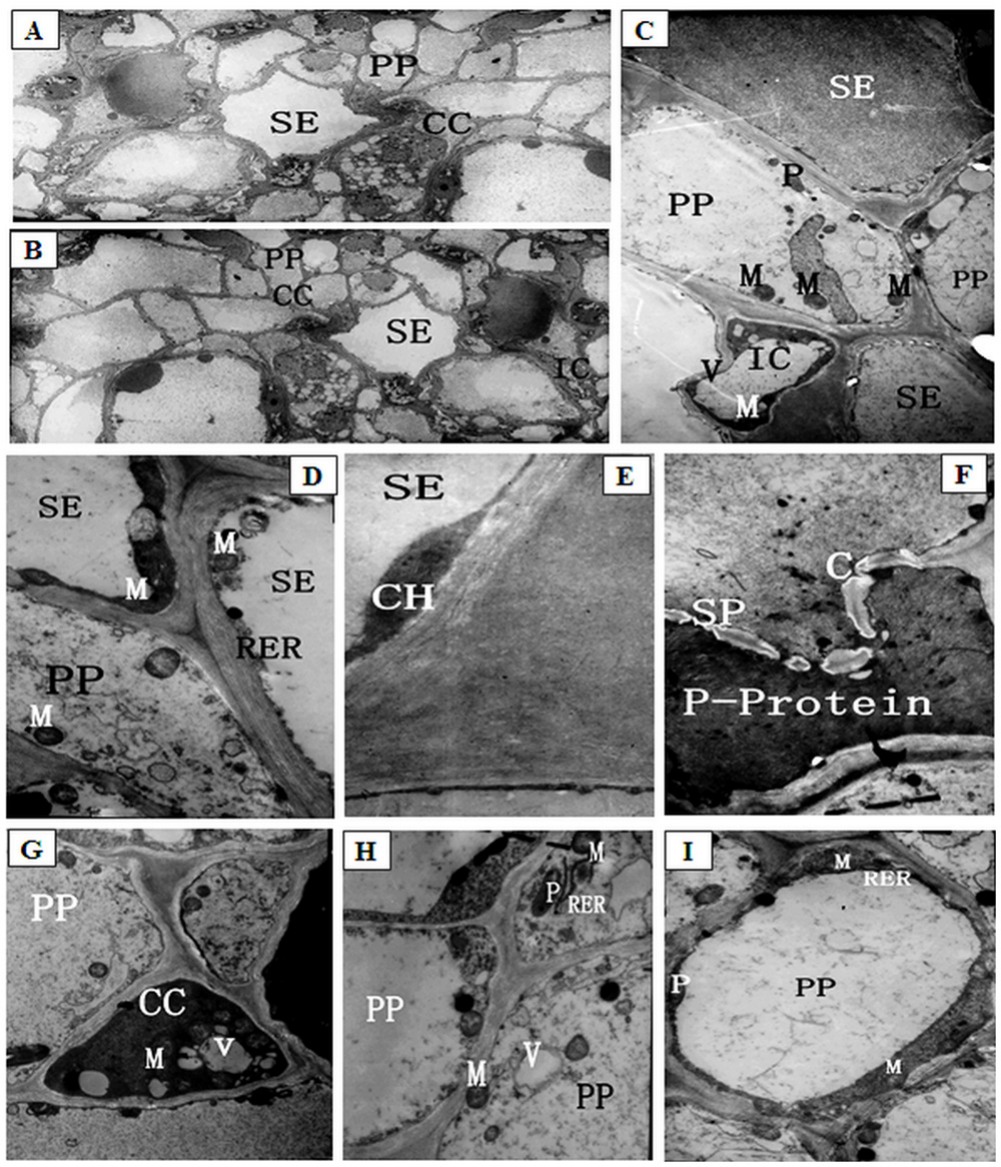
The ultrastructure of phloem in lateral branches of melons before low night temperature treatments. (A) Overall structure of phloem in lateral branches, ×2000; (B) The major cells of phloem in lateral branches, ×4000; (C) The developed sieve element (SE), developing sieve element and intermediary cells (IC) of phloem in lateral branches, ×58000; (D) The SE ultrastructure of phloem in lateral branches, ×58000; (E) The chloroplasts (CH) in SE of phloem in lateral branches, ×58000; (F) The sieve pores (SP) in SE of phloem in lateral branches, ×58000; (G) The companion cells (CC) of phloem in lateral branches, ×58000; (H) The phloem parenchyma cells (PP) of phloem in lateral branches, ×58000; (I) The phloem parenchyma cells (PP) becoming to sieve element of phloem in lateral branches, ×36000. SE: sieve tube element; CC: companion cells; IC: intermediary cells; PP: parenchyma cells; CH: chloroplast; N: nucleolus; V: vacuoles; P: plastids; M: mitochondria; RER: rough endoplasmic reticulum; P-protein: protein fiber system; SP: sieve plates.

The phloem in lateral branches of melons was mainly composed of sieve elements (SE), companion cells (CC), intermediary cells (IC) and phloem parenchyma cells (PP) (Fig 2A). The sieve elements and companion cells were the most important cells in the phloem. The sieve elements were rather large and formed channels for the transport of organic substances. The companion cells were much smaller than sieve elements, and their physiological functions were closely associated with those of sieve elements [20]. The phloem parenchyma cells were smaller than sieve elements but larger than companion cells and were scattered throughout the phloem (Fig 2B).

The fully mature sieve elements (SE) in the phloem of vascular bundles in the lateral branches were hollow conducting cells (Fig 2C). At early stages of development, sieve elements contain various cellular components. However, as the elements differentiate, the vacuolar membrane and a large amount of cytoplasm begin to disintegrate. Fully mature sieve elements presented an integrated plasma membrane, corrugated cell walls and no nucleus or vacuoles. These mature elements retained a small number of organelles, including mitochondria (M), plastids (P), rough endoplasmic reticulum (RER) and a protein fiber system (P-protein). The preserved protoplast was distributed along the periphery of the cell walls. Mitochondria were spherical, with a complete membrane structure and many cristae. The endoplasmic reticulum was strip-shaped and had numerous ribosomes attached to its surface. P-proteins appeared fibrous and were present in sieve elements. Chloroplasts (CH) were diamond or oval shaped. Chloroplast grana lamellae and interstitial lamellae were clearly visible and regularly arranged (Fig 2D and 2E). The transverse walls between two adjacent sieve elements formed sieve plates (SP) containing wide sieve pores. These pores develop from plasmodesmata and function to connect the two adjacent sieve elements. The plasmodesmata remaining in the sieve pore is termed the connecting strand. A small amount of callose had accumulated around the connecting strands along the periphery of the sieve pores (C-bright ring surrounding sieve pores). Some P-protein fibers were also found at sieve pores (Fig 2F).

Companion cells (CC) were located in close proximity to sieve elements and formed sieve element-companion cell complexes. These companion cells contained strongly stained, dense cytoplasm and numerous organelles, e.g., mitochondria and plastids (no starch). The companion cells also contained a large nucleus and a small number of vacuoles (V) (Fig 2G). It was worthy to note that the other type of companion cells in melons was special companion cells, referred to as “intermediary cells” (IC) (Fig 2C), which contained a large central vacuole, with the protoplast concentrated in the periphery of the cell cavity. The cytoplasm of these intermediary cells was dense, strongly stained. These cells were located next to sieve elements and played a role in the transport of substances [20].

Parenchyma cells (PP) in the phloem of vascular bundles in the lateral branches of melons were larger than companion cells. Phloem parenchyma cells were found around sieve elements. Compared with companion cells, the cytoplasm of parenchyma cells was rather sparse. In addition, these cells contained a relatively large, round or irregularly shaped nucleus and were rich in organelles such as spherical mitochondria and rod-shaped chloroplasts. Some endoplasmic reticulum was also present in phloem parenchyma cells (Fig 2H).

A subset of the parenchyma cells were in the process of differentiating into sieve elements. In these differentiating parenchyma cells, vacuoles underwent fusion to form larger vacuoles. A filamentous structure, likely composed of P-proteins, was found in the vacuoles. In the nuclei, the chromatin had begun to condense and aggregate towards the edge. The perinuclear space became enlarged, and the vacuoles gradually occupied most of the cell volume, with vacuolar fusion occurring at areas of contact between vacuoles. Small vesicles formed at the edge of the vacuoles, trapping cytoplasm inside, where it slowly disintegrated. The vacuoles then started to lose membrane integrity. Multi-membranous structures formed in some vacuoles, with a small amount of cytoplasm trapped within. The vacuolar membrane eventually broke down completely, and the clear boundary between the vacuoles and the cytoplasm disappeared. The cytoplasm then became rather sparse, leaving small floccules in the cells. The volume of the nucleus decreased, and apoptotic bodies formed. Ultimately, the nucleus completely disintegrated, and the cytoplasm nearly disappeared. Some parenchyma cells retained a small amount of membrane structure containing protein bodies (Fig 2C, 2H and 2I).

### Effects of low night temperature treatments on the phloem ultrastructure in lateral branches of melons

**Fig 3:**
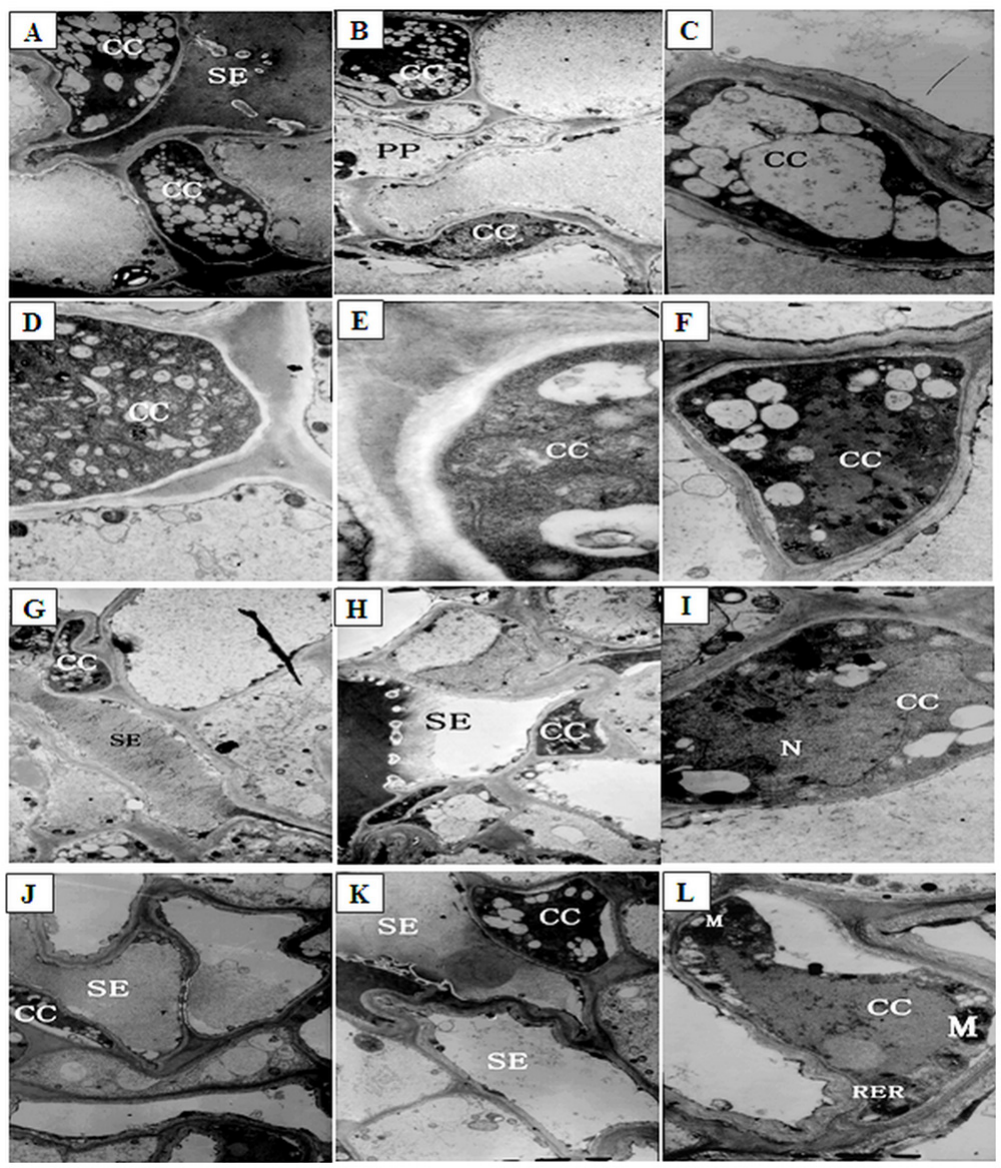
The ultrastructure of companion cells (CC) of phloem in lateral branches of melons after low night temperature treatments. (A-F) The CC of phloem in lateral branches of control plants (A and B: ×19000; C, D, E, and F: ×58000); (G-I) The CC of phloem in lateral branches after low night temperature treatment at12°C for 12 d (G and H: ×19000; I: ×58000); (J-L) The CC of phloem in lateral branches after low night temperature treatment at 9°C for 12 d (J and K: ×19000; L: ×58000). SE: sieve tube element; M: mitochondria; RER: rough endoplasmic reticulum.

Under normal temperature conditions, some of companion cells (CC) contained a large number of small vacuoles (Fig 3A and 3B). Vacuolar fusion occurred between the small vacuoles, generating large vacuoles that occupied most of the cellular space (Fig 3C). These cells showed strong electron staining and contained dense cytoplasm. Some of companion cells contained numerous smaller vacuoles and showed rather weak electron staining. The cell walls were slightly degraded (Fig 3D and 3E). Other companion cells contained only a handful of small vacuoles but also showed weak electron staining. The density of the cytoplasm was rather low (Fig 3B), and the cells appeared to contain large amounts of floccules (Fig 3F).

After 12 d of low night temperature treatment at 12°C, about 59% companion cells exhibited strong electron staining. These cells contained dense cytoplasm and numerous small vacuoles (Fig 3G). Slight plasma membrane invagination occurred in about 38% of these cells (Fig 3H) (Table 1). Some of the companion cells showed weaker electron staining and contained a large number of smaller vacuoles. About 41% cells contained slightly degraded cell walls and significantly deformed nuclei (Fig 3I). After 12 d of low night temperature treatment at 9°C, 99% companion cells showed notable plasma membrane invagination (Fig 3J–3L; S1, S2 and S3 Figs).

**Fig 4:**
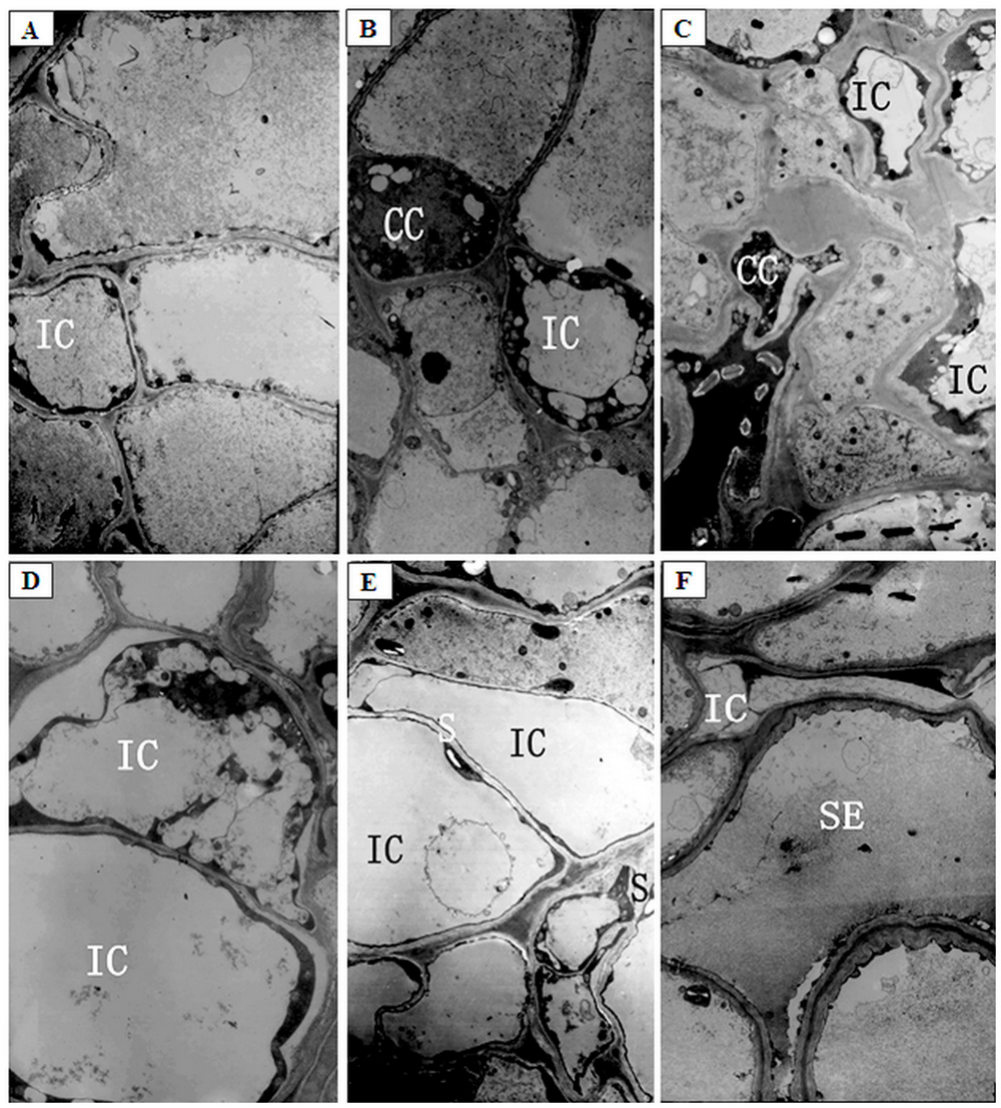
The ultrastructure of intermediary cells (IC) of phloem in lateral branches of melons after low night temperature treatments. (A, B) The IC of phloem in lateral branches of control plants, ×19000; (C) The IC of phloem in lateral branches after low night temperature treatment at 12°C for 12 d, ×19000; (D-F) The IC of phloem in lateral branches after low night temperature treatment at 9°C for 12 d (D: ×29000; E and F: ×19000). CC: companion cells; IC: intermediary cells; SE: sieve tube element; S: starch grain.

The number of intermediary cells (IC) decreased, from 50 to 25% in, as plants were growing under normal temperature conditions, most likely due to the continuous differentiation of intermediary cells into sieve elements. A cavity was formed at the center of all intermediary cells, and the cytoplasm was concentrated at the edge, forming a strongly stained, thin annular ring (Fig 4A). These intermediary cells contained a large number of vacuoles (Fig 4B). After 12 d of low temperature treatment at 12°C, the cytoplasm decreased by 50% and formed a more strongly stained, thinner annular ring around the edges of 96% intermediary cells, and many vacuoles were still observed or formed the central vacuole (Fig 4C) (Table 1; S4, S5, S6 and S7 Figs). After 12 d of low temperature treatment at 9°C, about 99% intermediary cells developed plasma membrane invaginations (Fig 4D and 4E). The majority of organelles in those cells were gradually degrading, and the central vacuole expanded (Fig 4D and 4F). Other intermediary cells might have become new sieve elements. The cytoplasm formed a strongly stained, thinner annular ring at the edges of intermediary cells, and the number of starch grains increased by 20% in these cells (Fig 4D and 4E).

**Fig 5:**
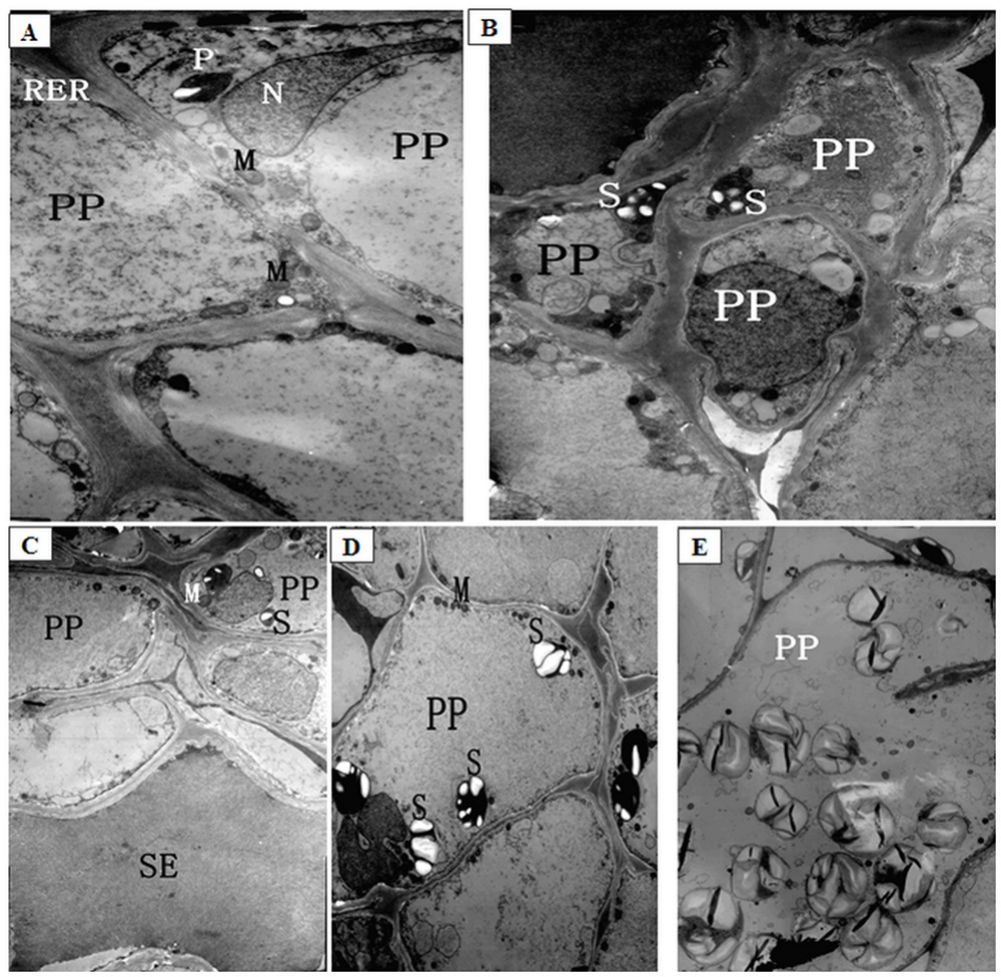
The ultrastructure of parenchyma cells (PP) of phloem in lateral branches of melons after low night temperature treatments. (A) The PP of phloem in lateral branches of control plants, ×36000; (B, C) The PP of phloem in lateral branches after low night temperature treatment at 12°C for 12 d, ×19000; (D) The PP of phloem in lateral branches after low night temperature treatment at 9°C for 12 d, ×19000; (E) The PP of phloem in lateral branches after low night temperature treatment at 9°C for 12 d, ×58000. PP: parenchyma cells; M: mitochondria; RER: rough endoplasmic reticulum; S: starch grain.

Under normal temperature conditions, the cytoplasm was sparse in phloem parenchyma cells (PP). Mitochondria and chloroplasts were concentrated around the edges of these cells, and little endoplasmic reticulum was present (Fig 5A). Compared with control plants (100%), some starch grains were present in almost all of parenchyma cells and their number was increased to 413% in total cells, and the number of mitochondria decreased by 5% after 12 d of low night temperature treatment at 12°C (Fig 5B and 5C). However, after 12 d of low night temperature treatment at 9°C, a large amount of starch grains appeared in the chloroplasts and the number was increased by 628% in total cells, the number of mitochondria decreased by 15% (Table 1). The remaining mitochondria was concentrated at the edges of the cells (Fig 5D and 5E).

### Effects of low night temperatures on the RFO contents in lateral branches and carpopodiums

**Fig 6:**
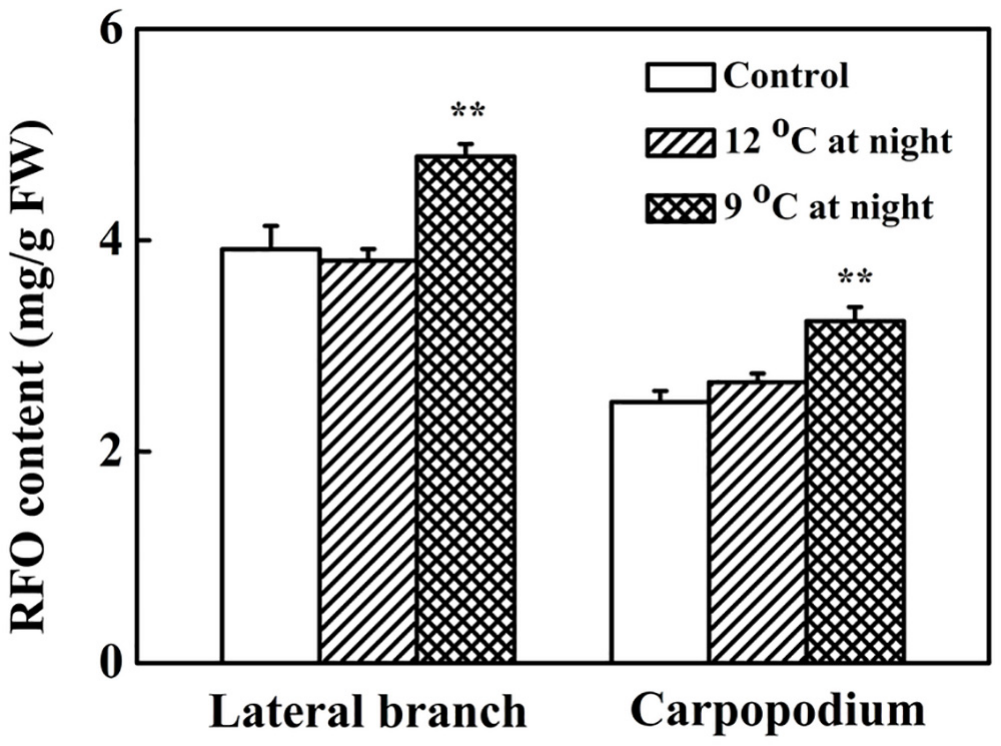
The raffinose family oligosaccharide (RFO) contents in vascular bundle of lateral branches and carpopodiums of melons after low night temperature treatments for 12 days. After 12-day treatments at 15°C (control night temperature) or 12°C or 9°C low night temperature, RFO contents were measured in the lateral branches and carpopodiums. Values are the means ±SD of three replicates (with each replicate being a pool of 3 plants); and ** P<0.01 compared to control.

Low night temperature treatments significantly affected the RFO content in lateral branches (Fig 6). Compared with the control, low night temperature treatment at 9°C for 12 d significantly increased the RFO content by 22.36% (P<0.01, P = 0.00346). In contrast, low night temperature treatment at 12°C did not induce significant changes in the RFO content.

Low night temperature treatments had a greater impact on the RFO content in carpopodiums (Fig 6). After 12 d of low night temperature treatment at 9°C, the carpopodiums had a RFO content of 3.23 mg/g, about 30.77% increase compared with control (P < 0.01, P = 0.00291). However, the RFO content in carpopodiums was not significantly affected by the low night temperature treatment at 12°C. These results indicated that low night temperature treatment at 9°C led to a large accumulation of RFO in carpopodiums, whereas the 12°C treatment had no such adverse effects.

## Discussion

As the main function of plant phloem is the transport of organic nutrients, primarily sugar [4,21], changes in the ultrastructure of phloem are closely associated with the sugar transport capacity in phloem. Low temperature stress can induce changes in the ultrastructure of plant cells [22]. In the present study, TEM analyses showed that low night temperature treatments, especially the 9°C treatment, altered the ultrastructure of the phloem tissues in lateral branches, including plasma membrane invaginations (being the most obvious change) in companion cells and intermediary cells, expanded central vacuole in intermediary cells, increased amounts of starch grains in phloem parenchyma cells, and fewer mitochondria in phloem parenchyma cells.

Plasma membrane invagination was observed being obvious in companion and intermediary cells following low night temperature treatment at 9°C. Plasma membrane invagination has been observed in wheat under low temperature stresses [23]. The physiological significance of this phenomenon lies in the fact that plasma membrane invagination leads to an apparent expansion of plasma membrane surface area. This, in turn, increases the total surface area available for metabolite exchange with cytoplasma and cell organelles. Plasma membrane invagination thus represents another adaptive response of phloem tissues to low temperature stresses. This is similar to the results form Venzhik [23] and Lütz [24].

Mitochondria are particularly sensitive to low temperatures, though the plasma membrane is the primary site of cold injury [25–27]. Mitochondria is the site of cellular energy conversion and energy production. Low night temperature treatment at 9°C resulted in a notable reduction about 15% in the number of mitochondria in phloem parenchyma cells located in the phloem of vascular bundles in lateral branches of melons. A low number of mitochondria may affect cellular energy supply and this may possibly be related with limited RFO transport under low temperature conditions.

Low night temperature treatments also increased the number of starch grains in chloroplasts of phloem parenchyma cells. One potential explanation of starch accumulation would be that, due to the low night temperature, sink strength of developing fruit and other sink parts of the plant is reduced as a result of decreased metabolic activity and thus there is less demand for carbon to support metabolism and growth. This would then lead to starch storage from carbon.

Vacuoles are important components of the plant protoplasts. They are surrounded by vacuolar membranes and filled with dissolved substances, including hydrolytic enzymes. Once released from the vacuoles, these enzymes hydrolyze certain cellular components [28,29]. Low night temperature (9°C) caused increased numbers of vacuoles in intermediary cells, which later fused to a central vacuole. When the volume of a vacuole reached a certain level, vacuolar membrane fission or boundary blurring occurred. In this condition, substances with a relatively low electron density were observed near the vacuolar membrane or inside the vacuoles. It was clear that the release of these substances was closely related to organelle disintegration. Indeed, organelles began to disintegrate following vacuolar membrane fission or the blurring of the vacuolar membrane boundary. We believed that the substances with a low electron density were enzymes that promote organelle disintegration. Therefore, an increase of vacuoles in the companion cells might be the first indication of the onset of organelle degradation. This was similar to the report of Gamalei that vesicles in ICs of minor veins collapsed at low temperatures due to a presumptive destabilization of the cytoskeleton [30]. This collapse would explain the cold sensitivity of symplastic phloem loading as the vesicles were speculated to be a part of an endoplasmic continuum extending from mesophyll cell to minor-vein sieve elements that was involved in photo assimilate transfer [30]. In our previous studies, it was found that low night temperature treatment at 9°C resulted in the decline of carbohydrate metabolism in leaves. It also caused small fruits and a low content of sugar in fruits [15]. Based on these results and those from the current study, it can be speculated that the phloem transport may be limited, and that the changes in phloem ultrastructure may be related to the reduced phloem transport.

Our results also showed that low night temperature treatment at 9°C resulted in an increase in the RFO accumulation in lateral branches and carpopodiums. Popov et al. showed that hypothermia inhibited active transport in vascular bundles by affecting metabolic activity [31]. Caskill et al used ^14^CO_2_ as a tracer to evaluate the ^14^C content of phloem sap in purple mullein and showed that phloem transport velocity declined at low temperatures [32]. It is possible that the accumulation of RFO in lateral branches and carpopodiums in response to low night temperature observed in the present study reflect the repressed phloem transport. Future studies are required to examine whether low night temperature stress may affect phloem transport. Interestingly, in the treatment of 9°C at night the increase in RFO content in the carpopodiums was larger than that in lateral branches, while the amount of RFO was lower in carpopodiums compared with lateral branches. This indicated that carpopodiums were more sensitive to low night temperature than lateral branches, and that the RFO in carpopodiums might be degraded to sucrose or hexose in fruits, a possibility that needs to be confirmed in future studies.

## Conclusions

Low night temperature treatments at 9°C and 12°C altered the ultrastructure of cells of the transport structure (phloem) to varying degrees, with the changes at 9°C being more obvious. The ultrastructural changes included plasma membrane invagination in companion and intermediary cells; the presence of central vacuole in intermediary cells; the appearance of large numbers of starch grains, and reduced numbers of mitochondria in phloem parenchyma cells. In addition, RFO also accumulated in the vascular bundles of lateral branches and fruit carpopodiums. The changes described above represent adaptive responses of melons to low temperature stresses. However, these changes also result in a shortage of the cellular energy required for phloem transport and cellular damage, neither of which is conducive to phloem transport.

## Supporting Information

S1 Fig The companion cells (CC) of phloem in lateral branches after low night temperature treatment at 9°C for 12 d. NO plasma membrane invagination in companion cells (×36000).
(PDF)

S2 Fig The companion cells (CC) of phloem in lateral branches after low night temperature treatment at 9°C for 12 d. NO plasma membrane invagination in companion cells (×58000).
(PDF)

S3 Fig The companion cells (CC) of phloem in lateral branches after low night temperature treatment at 9°C for 12 d. NO plasma membrane invagination in companion cells (×19000).
(PDF)

S4 Fig The intermediary cells (IC) of phloem in lateral branches after low night temperature treatment at 9°C for 12 d. NO plasma membrane invagination in intermediary cells (×19000).
(PDF)

S5 Fig The intermediary cells (IC) of phloem in lateral branches after low night temperature treatment at 12°C for 12 d. NO expanded central vacuole and strongly stained and thinner annular rings in intermediary cells (×14000).
(PDF)

S6 Fig The intermediary cells (IC) of phloem in lateral branches after low night temperature treatment at 9°C for 12 d. NO expanded central vacuole and strongly stained and thinner annular rings in intermediary cells (×19000).
(PDF)

S7 Fig The intermediary cells (IC) of phloem in lateral branches after low night temperature treatment at 9°C for 12 d. NO expanded central vacuole and strongly stained and thinner annular rings in intermediary cells (×19000).
(PDF)
