# Supplementary material for: Low Night Temperature Affects the Phloem Ultrastructure of Lateral Branches and Raffinose Family Oligosaccharide (RFO) Accumulation in RFO-Transporting Plant Melon (Cucumismelo L.) during Fruit Expansion
Source: PLoS One. 2016 Aug 8;11(8):e0160909. doi: 10.1371/journal.pone.0160909 (PMC4976869; doi:10.1371/journal.pone.0160909)
Supplement: S2 Fig — NO plasma membrane invagination in companion cells (×58000). (PDF) [file pone.0160909.s002.pdf]

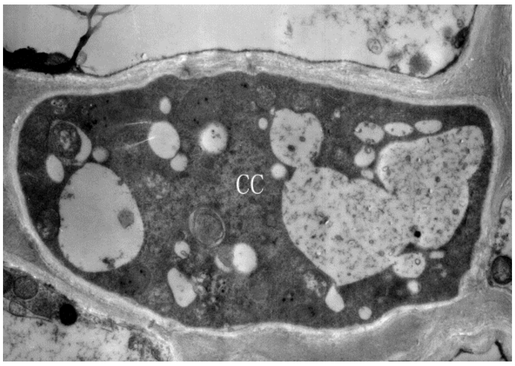

S2 Fig. The companion cells (CC) of phloem in lateral branches after low night temperature treatment at 9 ℃ for 12 d. NO plasma membrane invagination in companion cells (×58000).
